# The RNA-binding protein AUF1 facilitates Akt phosphorylation at the membrane

**DOI:** 10.1016/j.jbc.2022.102437

**Published:** 2022-08-27

**Authors:** Mei-Ling Li, Aparna Ragupathi, Nikhil Patel, Tatiana Hernandez, Jedrick Magsino, Guy Werlen, Gary Brewer, Estela Jacinto

**Affiliations:** Department of Biochemistry and Molecular Biology, Rutgers Biomedical Health Sciences-Robert Wood Johnson Medical School, Piscataway, New Jersey, USA

**Keywords:** mTOR, mTORC2, Akt, AUF1, hnRNP D, RNA-binding protein, glutamine, AUF1, ARE/poly(U)-binding/degradation factor 1, FBS, fetal bovine serum, GFAT1, glutamine fructose-6-phosphate amidotransferase 1, GSK3, glycogen synthase kinase 3, HBP, hexosamine biosynthesis pathway, hnRNP D, heterogeneous nuclear ribonucleoprotein D, HSP, high-speed pellet, IP, immunoprecipitation, KD, kinase-dead, mRNP, messenger ribonucleoprotein, mTOR, mammalian target of rapamycin, mTORC, mTOR complex, PAR-CLIP, photoactivatable ribonucleoside-enhanced crosslinking and immunoprecipitation, PH, pleckstrin homology, PIP3, phosphatidylinositol 3,4,5-trisphosphate, qRT–PCR, quantitative RT–PCR, RBP, RNA-binding protein, TCGA, The Cancer Genome Atlas

## Abstract

Mammalian target of rapamycin (mTOR), which is part of mTOR complex 1 (mTORC1) and mTORC2, controls cellular metabolism in response to levels of nutrients and other growth signals. A hallmark of mTORC2 activation is the phosphorylation of Akt, which becomes upregulated in cancer. How mTORC2 modulates Akt phosphorylation remains poorly understood. Here, we found that the RNA-binding protein, AUF1 (ARE/poly(U)-binding/degradation factor 1), modulates mTORC2/Akt signaling. We determined that AUF1 is required for phosphorylation of Akt at Thr308, Thr450, and Ser473 and that AUF1 also mediates phosphorylation of the mTORC2-modulated metabolic enzyme glutamine fructose-6-phosphate amidotransferase 1 at Ser243. In addition, AUF1 immunoprecipitation followed by quantitative RT–PCR revealed that the mRNAs of Akt, glutamine fructose-6-phosphate amidotransferase 1, and the mTORC2 component SIN1 associate with AUF1. Furthermore, expression of the p40 and p45, but not the p37 or p42, isoforms of AUF1 specifically mediate Akt phosphorylation. In the absence of AUF1, subcellular fractionation indicated that Akt fails to localize to the membrane. However, ectopic expression of a membrane-targeted allele of Akt is sufficient to allow Akt-Ser473 phosphorylation despite AUF1 depletion. Finally, conditions that enhance mTORC2 signaling, such as acute glutamine withdrawal, augment AUF1 phosphorylation, whereas mTOR inhibition abolishes AUF1 phosphorylation. Our findings unravel a role for AUF1 in promoting membrane localization of Akt to facilitate its phosphorylation on this cellular compartment. Targeting AUF1 could have therapeutic benefit for cancers with upregulated mTORC2/Akt signaling.

Cells respond to the availability of nutrients by controlling gene expression at the level of both transcription and translation. Mammalian target of rapamycin (mTOR) plays a central role in sensing the nutritional status of the cell and triggers a cascade of intracellular signaling that ultimately promotes anabolic metabolism, growth, and proliferation (1, 2, 3). Deregulation of mTOR signaling occurs in many diseases, including cancer, diabetes, autoimmunity, and neurological disorders. Dampening mTOR signals is a promising strategy for the treatment of these diseases and to improve health span (4, 5). mTOR forms two distinct protein complexes, mTOR complex 1 (mTORC1) and mTOR complex 2 (mTORC2). Many studies have revealed how mTORC1, which is sensitive to rapamycin, is regulated by nutrients and how intracellular signaling molecules mediate its functions. In contrast, how mTORC2 is regulated and the identity of its downstream effectors remain poorly understood (6).

One of the hallmarks of increased mTORC2 activation is the allosteric phosphorylation of Akt at the hydrophobic motif site, Ser473 (7, 8, 9). Upon growth factor signaling, PI3K becomes activated leading to increased phosphatidylinositol 3,4,5-trisphosphate (PIP3) levels in the membrane. Enhanced PIP3 levels attract signaling molecules with the pleckstrin homology (PH) domain, such as Akt, to the membrane where it becomes phosphorylated at the activation loop site, Thr308, by PDK1 and at Ser473 *via* an mTORC2-dependent mechanism (8, 9, 10, 11). Both PDK1 and mTORC2 have been found to localize on the membrane. PDK1 and the mTORC2 components SIN1 and possibly rictor each harbor a PH domain that could deliver them to the membrane to promote Akt activation (12, 13, 14). In addition to phosphorylation at Ser473, mTORC2 also mediates Akt phosphorylation at the “turn motif” site, Thr450 (15, 16). Unlike Ser473, the phosphorylation of this site is PI3K independent and occurs during translation (17). Thr450 phosphorylation is sensitive to glucose deprivation and acute ATP depletion (18).

We and others have shown that mTORC2 is activated during withdrawal of nutrients such as glucose or glutamine (19, 20). mTORC2 responds to the levels of intracellular glutamine metabolites during glucose or glutamine starvation (19). Its activation is important to maintain flux through the hexosamine biosynthesis pathway (HBP) *via* modulation of the rate-limiting enzyme of the *de novo* HBP, glutamine fructose-6-phosphate amidotransferase 1 (GFAT1). These findings reveal that mTORC2 responds not only to the presence of growth signals but also to nutrient fluctuations in order to restore metabolic homeostasis.

Accumulating evidence supports that the expression levels of mTORC2 components could modulate its activity, and their deregulation occurs in cancer (21, 22, 23, 24, 25, 26, 27, 28). Given the central role of mTORC2 in modulating metabolism and cell proliferation, a better understanding of the different mechanisms of mTORC2 regulation at the level of translation control could provide insights on more specific therapeutic strategies in cancer. Using photoactivatable ribonucleoside-enhanced crosslinking and immunoprecipitation (PAR-CLIP) transcriptome-wide screen, the mRNAs encoding mTORC2 components mTOR, rictor, and the HBP enzyme GFAT1 were among the putative targets of the RNA-binding protein (RBP) AUF1 (ARE/poly(U)-binding/degradation factor 1; also known as heterogeneous nuclear ribonucleoprotein D [hnRNP D]) (29). AUF1 has been linked to the regulation of mRNA decay, translation, and miRNA (30, 31, 32). It can either stabilize or destabilize mRNAs, but it remains obscure how AUF1 can perform distinct functions on diverse RNA targets (33, 34, 35, 36). It has strong affinity for AU-rich RNA sequences, but recent studies indicate that it primarily recognizes U-/GU-rich sequences in mRNAs and noncoding RNAs (29). AUF1 consists of four isoforms that are generated by alternative splicing of a common pre-mRNA (37). The isoforms display varying affinities with their RNA targets (36). Based on the emerging role of mRNA regulation of mTORC2 components in modulating mTORC2 activity (38, 39, 40), we investigated how AUF1 might function in modulating mTORC2 signaling and function. We found that AUF1 is required for the phosphorylation of Akt at the sites regulated by PDK1 and mTORC2. This is surprising given the known role of AUF1 in modulating mRNA stability and translation of its targets. We demonstrate that AUF1 mediates the membrane localization of Akt where it is phosphorylated in response to growth signals. We also found that AUF1 is modulated by signals that enhance mTORC2 activation. Our findings identify a role for AUF1 in regulating mTORC2 signaling.

## Results

### Phosphorylation of Akt is abolished upon AUF1 knockdown

**Figure 1:**
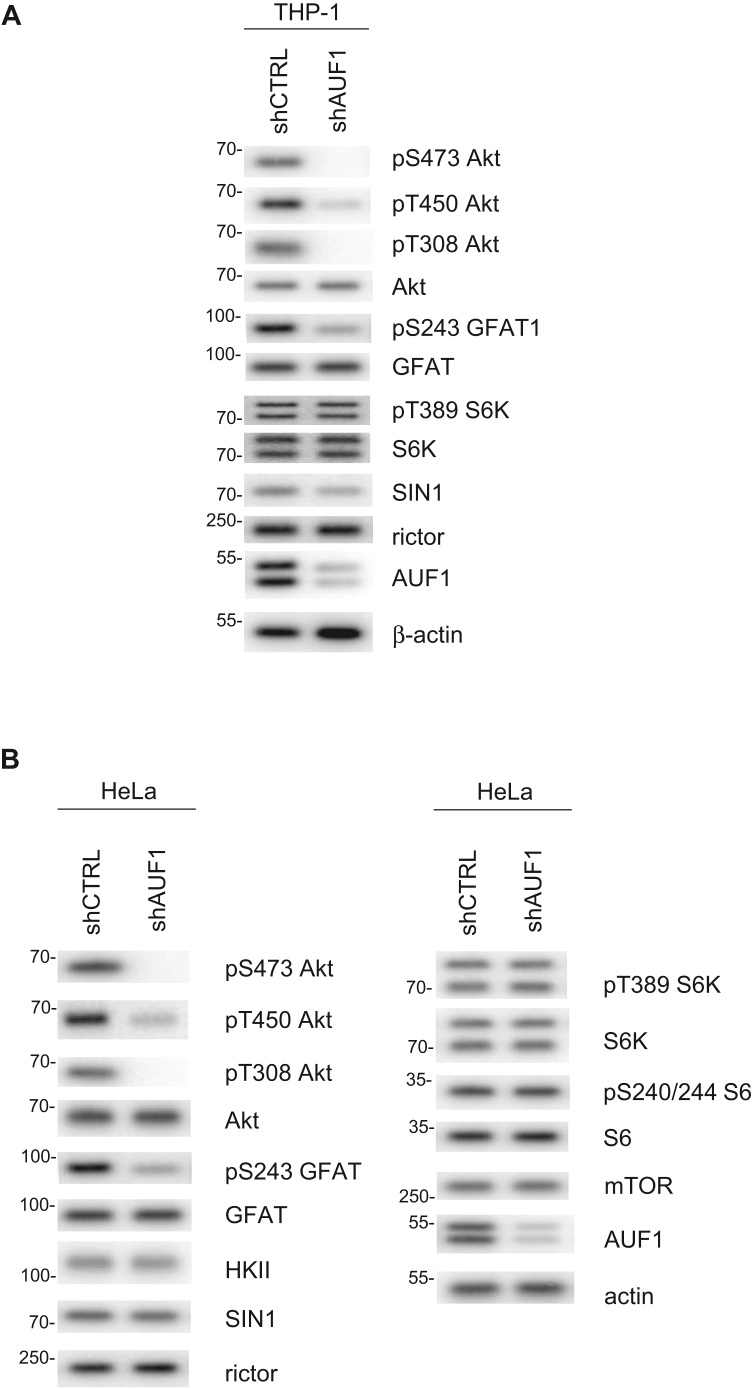
**Phosphorylation of Akt is abolished upon AUF1 knockdown.***A*, THP-1 cells were stably transfected with plasmids expressing either control shCTRL or shAUF1. Cell extracts were subjected to SDS-PAGE and immunoblotting. *B*, HeLa cells were transiently transfected with control shCTRL or shAUF1 plasmids. After 2 days, HeLa cells were harvested, and cellular extracts were subjected to SDS-PAGE and immunoblotting. Phosphorylated or total proteins are indicated. AUF1, ARE/poly(U)-binding/degradation factor 1.

In a transcriptome-wide screen to analyze genes that are regulated by AUF1, components of the mTOR pathway including mTOR and rictor were identified by PAR-CLIP analysis (29). We therefore hypothesized that AUF1 could modulate mTOR signaling. Using the human promonocytic cell line THP-1, we knocked down AUF1 by stable transfection of an shAUF1 expression plasmid and analyzed readouts of both mTORC1 and mTORC2 signaling. Strikingly, phosphorylation of the mTORC2 target sites in Akt, Ser473 and Thr450, was diminished in AUF1-depleted cells but not in shCTRL plasmid-transfected cells (Fig. 1*A*). The phosphorylation of the activation loop site in Akt, Thr308, which is mediated by PDK1 was also abolished upon AUF1 knockdown. We have recently shown that the key metabolic enzyme of the HBP, GFAT1 (aka GFPT1) was modulated by mTORC2. Interestingly, GFPT1 mRNA was also identified in the PAR-CLIP analysis (29). We therefore examined its phosphorylation and found that it was also diminished upon AUF1 knockdown. In contrast, phosphorylation of the mTORC1 target S6K (at Thr389) was not affected. Consistent with a defective mTORC2 signaling, there was a slight decrease in SIN1 but not rictor expression in THP-1 cells.

We also knocked down AUF1 in HeLa cells by transient transfection with the shAUF1 expression plasmid or siAUF1 and found that the phosphorylation of Akt was diminished in AUF1-depleted cells but not in plasmid shCTRL- or si-scrambled transfected cells (Figs. 1*B* and S1, respectively). This indicates that the impact of shAUF1 on Akt phosphorylation was not because of an off-target effect of the shAUF1 sequence or an indirect effect of shRNA expression.

GFAT1 phosphorylation was also reduced upon AUF1 depletion in HeLa cells (Fig. 1*B*). In contrast, the phosphorylation of the mTORC1 effectors, S6K and the ribosomal protein S6, was not altered. Interestingly, the expression of SIN1, rictor, and mTOR was not diminished when AUF1 expression was reduced. Furthermore, mTORC2 integrity was not compromised in AUF1-depleted cells, as both mTOR and rictor co-IP with SIN1 regardless of AUF1 expression levels (Fig. S2). Hence, the decrease in Akt phosphorylation during AUF1 knockdown in HeLa cells is likely because of a more direct effect on Akt, rather than mTORC2.

### AUF1 binds to Akt, GFAT1, and SIN1 mRNAs

**Figure 2:**
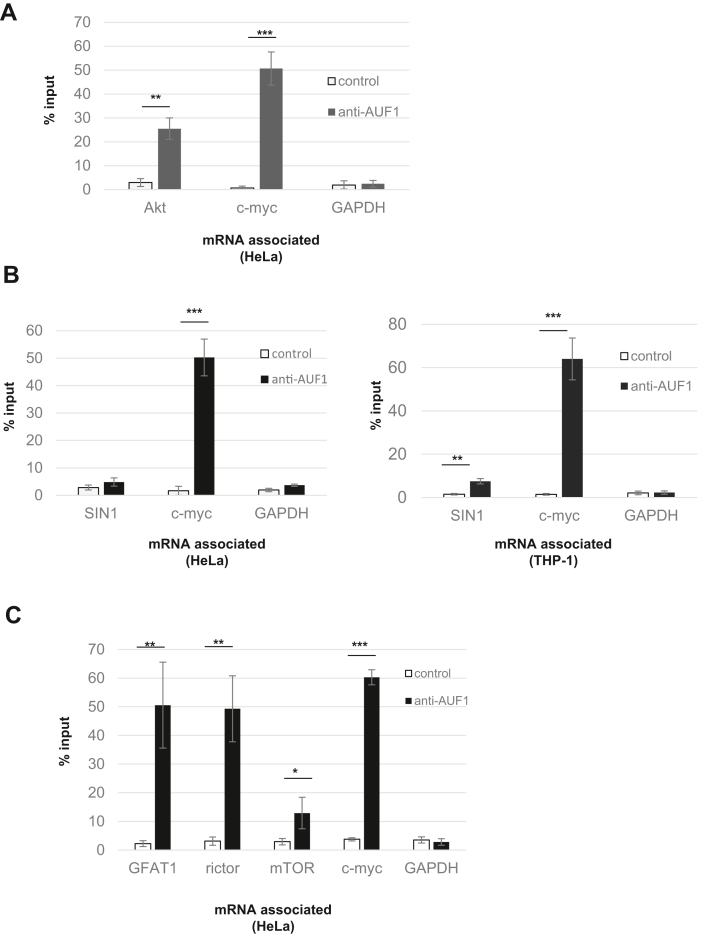
**AUF1 binds to Akt, SIN1, and GFAT1 mRNA.***A*–*C*, cellular lysates from THP-1 or HeLa cells were subjected to immunoprecipitation using control (nonimmune serum) or AUF1 antibody. qRT–PCR was then performed using RNA purified from immunoprecipitates and specific primers for Akt, SIN1, GFAT1, rictor, mTOR, c-Myc, and GAPDH. Comparisons of mRNA levels between control *versus* AUF1 antibody were performed using the unpaired *t* test with differences exhibiting *p* < 0.05 considered significant. n = 3 independent experiments; Error bars indicate SD. ∗*p* < 0.01, *∗∗p* < 0.001, and *∗∗∗p* < 0.0001. AUF1, ARE/poly(U)-binding/degradation factor 1; GFAT1, glutamine fructose-6-phosphate amidotransferase 1; mTOR, mammalian target of rapamycin; qRT–PCR, quantitative RT–PCR.

We next investigated if AUF1 binds to Akt mRNA. Using messenger ribonucleoprotein (mRNP) immunoprecipitation analysis, we immunoprecipitated AUF1 and then quantitated AUF1-bound transcripts by quantitative RT–PCR (qRT–PCR). Compared with precipitates using nonimmune serum, Akt mRNA was enriched in the AUF1 immunoprecipitates about fivefold, thus indicating that AUF1 forms an mRNP complex with Akt mRNA (Fig. 2*A*). As expected, c-myc mRNA robustly binds to AUF1, whereas GAPDH mRNA does not (41). Since SIN1 expression was diminished upon AUF1 knockdown in THP-1 but not HeLa cells, we also examined AUF1 association with SIN1 mRNA. Consistent with the slight decrease in expression of SIN1 in THP-1 cells, a discernible, but statistically significant, increase in association of SIN1 mRNA with AUF1 in THP-1 but not in HeLa cells occurred (Fig. 2*B*). Finally, since GFAT1 phosphorylation was also altered upon knockdown of AUF1, we then examined if its mRNA associates with AUF1. Consistent with the PAR-CLIP transcriptome-wide analysis (29), we confirmed that AUF1 binds to GFAT1 mRNA (Fig. 2*C*). Together, our findings indicate that AUF1 associates with mRNAs of Akt and other proteins that are linked to mTORC2 signaling.

### AUF1 facilitates Akt phosphorylation by mediating membrane localization

**Figure 3:**
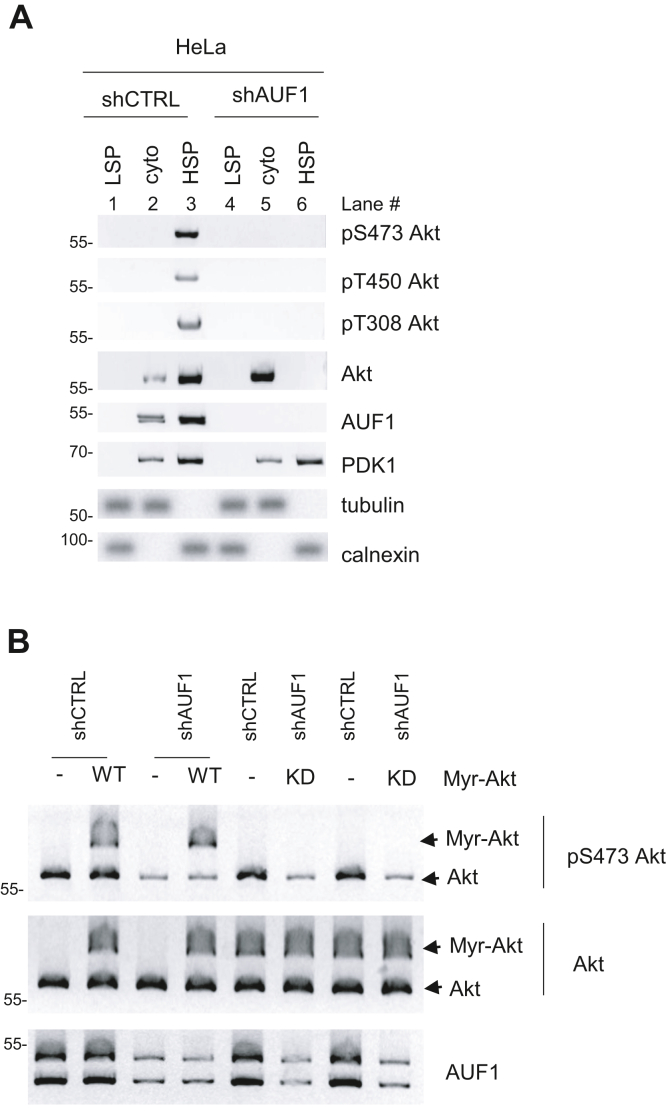
**AUF1 facilitates Akt phosphorylation by mediating membrane localization.***A*, extracts of HeLa cells that were transiently transfected with either control shCTRL or shAUF1 plasmids were fractionated *via* differential centrifugation. Cytosolic (cyto) or the membrane-containing *low-speed* pellet (LSP) or *high-speed* pellet (HSP) fractions were subjected to SDS-PAGE and immunoblotting. *B*, HeLa cells, transfected with shCTRL or shAUF1 plasmids together with either the vector control (−) or Myr-Akt (either WT or kinase dead [KD]) plasmids, were harvested and subjected to SDS-PAGE and immunoblotting. AUF1, ARE/poly(U)-binding/degradation factor 1.

Since Akt phosphorylation occurs at the membrane, we asked if AUF1 is involved in localizing Akt in this compartment. By cellular fractionation, we found that whereas Akt predominantly localizes to the membrane-containing high-speed pellet (HSP) fractions in the control (shCTRL) cells (Fig. 3*A*, lane 3), it was absent in this compartment (lane 6) and instead was mainly present in the cytosolic fractions in AUF1-knocked down cells (lane 5). Phosphorylated Akt (at Ser473, Thr450, and Thr308) was present only in the HSP fractions in the control cells (lane 3). The Akt that fractionated at the cytosolic fractions in AUF1-knocked down cells was unphosphorylated (lane 5). In contrast, PDK1 localization in the cytosol and HSP was not altered in the absence of AUF1 (lanes 2 and 3 *versus* lanes 5 and 6). These findings indicate that AUF1 facilitates the localization of Akt at the subcellular membrane-containing compartments that fractionate in the HSP. Furthermore, AUF1 is required for Akt phosphorylation at these compartments.

Since the aforementioned findings suggest that AUF1 is necessary for Akt phosphorylation at the membrane, we next asked whether AUF1 is still required for Akt phosphorylation once Akt is already at the membrane. To address this, we expressed the Myr-Akt fusion construct, which constitutively localizes Akt to the membrane (15) under AUF1-replete (shCTRL) or AUF1-deplete (shAUF1) conditions. When AUF1 expression was silenced, the phosphorylation of Myr-Akt at Ser473 remained similar to control cells, whereas the endogenous Akt had diminished phosphorylation (Fig. 3*B*). Overexpression of Myr-Akt with a kinase-dead (KD) mutation (15) prevented phosphorylation of Myr-Akt, confirming that the kinase activity of Akt is required for Ser473 phosphorylation (42). Together, these findings indicate that AUF1 mediates the membrane localization of Akt where it becomes phosphorylated at Ser473.

### p40 and p45 AUF1 isoforms specifically promote Akt phosphorylation

**Figure 4:**
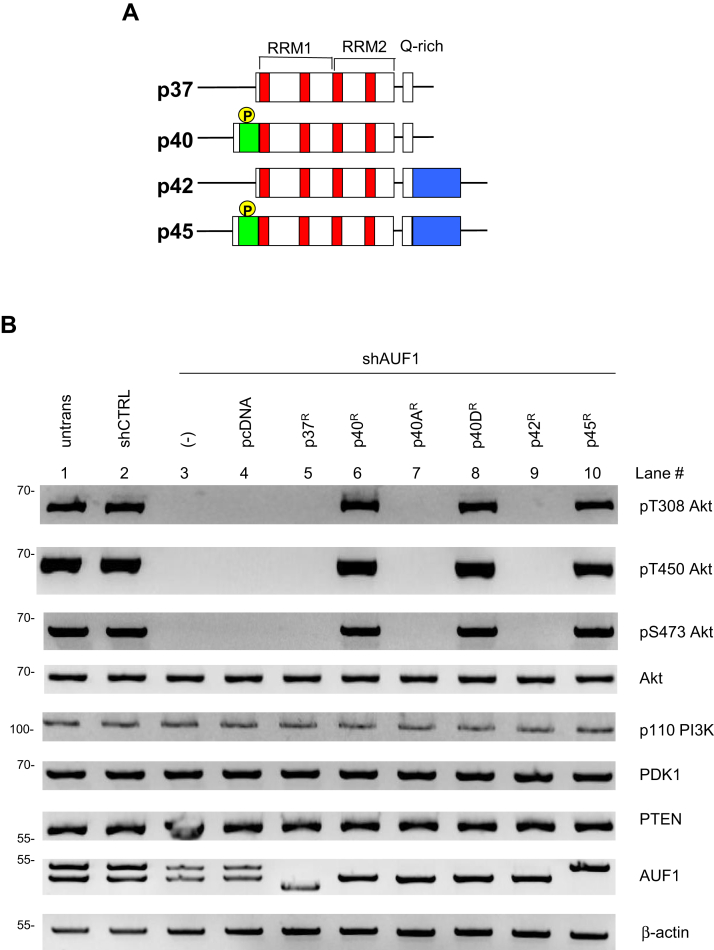
**p40 and p45 AUF1 isoforms specifically promote Akt phosphorylation.***A*, AUF1 has four different isoforms. The phosphorylated N-terminal region in p40 and p45 is indicated in *green*, phosphorylation (P) is indicated in *yellow*. The conserved RNP-2 and RNP-1 *boxes* within each RNA-recognition motif (RRM, *bracketed*) are indicated in *red*. The extended C terminus in p42 and p45 is indicated in *blue*. *B*, HeLa cells were transiently transfected with either shCTRL alone (lane 2) or shAUF1 plasmids without (−) or with the empty vector (pcDNA), or the indicated AUF1 WT or mutant expression plasmids (lanes 3–10). The “R” mutants are resistant to knockdown by shAUF1 because of silent mutations within the AUF1 coding region. “A” indicates the Ser(83,87)-to-Ala mutant, which is phosphodeficient, whereas “D” indicates Ser(83,87)-to-Asp mutant, which is phosphomimetic. Lysates of transfected cells were subjected to SDS-PAGE and immunoblotting. AUF1, ARE/poly(U)-binding/degradation factor 1; RNP, ribonucleoprotein.

AUF1 consists of four isoforms that are generated by alternative splicing of a common pre-mRNA. All isoforms contain the RNA recognition motif domains, RRM1 and RRM2 followed by a glutamine (Q)-rich domain (Fig. 4*A*). p40 and p45 isoforms have an additional 19-amino acid N-terminal region generated from alternatively spliced exon 2, whereas p42 and 45 isoforms harbor an extended C-terminal region generated from alternatively spliced exon 7. AUF1 is phosphorylated within the exon 2-encoded domain at Ser83, Ser87, and Thr91 (43, 44). This domain is present in p40 and p45 but not p37 and p42. Dephosphorylation of these sites is linked to induction of a more condensed RNA conformation on AUF1 substrates that could influence mRNA stability (43). We thus investigated which of these AUF1 isoforms might be required for Akt phosphorylation. Using HeLa cells in which endogenous AUF1 expression was reduced by transfection of the shAUF1 expression plasmid, we reconstituted expression of each individual AUF1 isoform in HeLa cells by transfecting a plasmid encoding the respective isoform that contained AUF1 sequences refractory to shAUF1 (designated by “R” in their names) because of silent mutations (45). Expression of p40^R^ and p45^R^, but not p37^R^ or p42^R^, enabled phosphorylation of Akt in shAUF1-expressing cells, suggesting that the additional N-terminal region that harbors the phosphoregulatory sites in AUF1 is required to mediate Akt phosphorylation (Fig. 4*B*, lanes 6 and 10). Expression of the phosphomimetic p40D^R^, in which both Ser83 and Ser87 were mutated to Asp, restored Akt phosphorylation (Fig. 4*B*, lane 8). By contrast, the phosphodeficient p40A^R^ mutant, harboring Ala instead of Ser, failed to restore Akt phosphorylation (Fig. 4*B*, compare lane 8 to lane 7). Western blot analysis of cell lysates confirmed comparable expression of each ectopically expressed AUF1 isoform (Fig. 4*B*, lanes 5–10). The expression of PI3K, PDK1, and PTEN was not altered by restoring expression of any of the AUF1 isoforms, supporting that the effect of these isoforms on Akt phosphorylation is likely not *via* alteration of expression of each of these Akt signaling modulators. Hence, p40 and p45 specifically regulate Akt phosphorylation.

### AUF1 is phosphorylated by signals that enhance mTORC2 signaling

**Figure 5:**
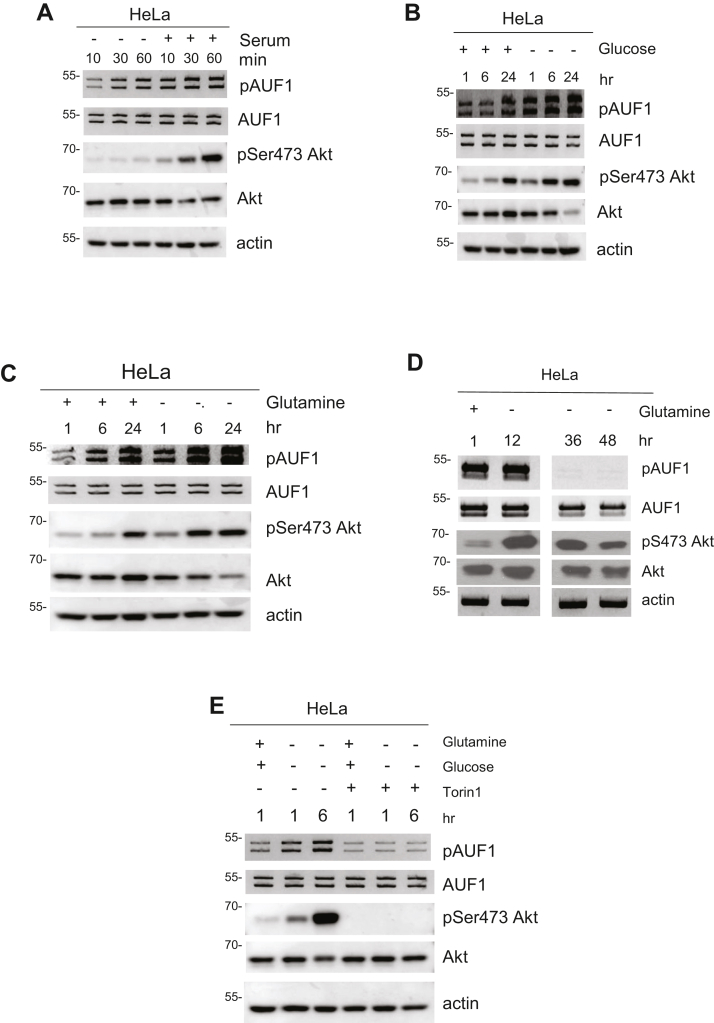
**AUF1 is phosphorylated by signals that enhance mTORC2 signaling.***A*, growing HeLa cells were resuspended in complete media lacking (−) or containing (+) FBS (1× serum) and then incubated at the indicated times prior to harvest. Cell lysates were processed for immunoblotting. *B*–*D*, growing HeLa cells were resuspended in media containing (+) or lacking (−) glucose (*B*) or lacking glutamine (*C* and *D*) for the indicated hours. Cell lysates were processed as in *A*. *E*, growing HeLa cells were resuspended in media containing (+) or lacking (−) glucose and glutamine. Torin1 (2 μM) or vehicle (−) was added as indicated, and cells were incubated for either 1 or 6 h. Cells were processed as in *A*. AUF1, ARE/poly(U)-binding/degradation factor 1; FBS, fetal bovine serum; mTORC2, mammalian target of rapamycin complex 2.

Since we found that phosphorylated p40 is necessary for Akt phosphorylation, we next examined how AUF1 phosphorylation could be modulated by signals that enhance mTORC2 signaling. First, we analyzed how serum stimulation, which increases mTORC2/Akt signaling, could affect AUF1 phosphorylation. Serum stimulation increased Akt-Ser473 phosphorylation robustly from 30 to 60 min and coincided with a discernible increase in AUF1 phosphorylation during these time points (Fig. 5*A*). We and others have also demonstrated that glucose limitation augments Akt phosphorylation (19, 20). Prolonged incubation (24 h) of HeLa cells in complete media enhanced Akt phosphorylation as well as AUF1 phosphorylation (Fig. 5*B*). Withdrawal of glucose from the media robustly increased AUF1 phosphorylation at all time points examined, similar to Akt phosphorylation. During acute glutamine withdrawal (up to 24 h in HeLa), Akt Ser473 phosphorylation remained robust, and this coincided with increased AUF1 phosphorylation (Fig. 5*C*). By 36 to 48 h glutamine starvation, Akt phosphorylation declined as we previously reported (19), and AUF1 phosphorylation was absent during these time points (Fig. 5*D*). Since Akt phosphorylation is abolished by mTORC2 inhibition, we therefore examined if AUF1 phosphorylation is also sensitive to this treatment. We used Torin1, which inhibits mTOR (both mTORC1 and mTORC2) and assessed its effect on AUF1 phosphorylation during combined glucose and glutamine withdrawal. Torin1 prevented the increase in AUF1 phosphorylation during glucose and glutamine starvation (Fig. 5*E*). As expected, it abolished Akt-Ser473 phosphorylation. These findings reveal that AUF1 phosphorylation is positively modulated by signals that enhance mTORC2 signaling.

### AUF1 expression is increased in liver tumors

**Figure 6:**
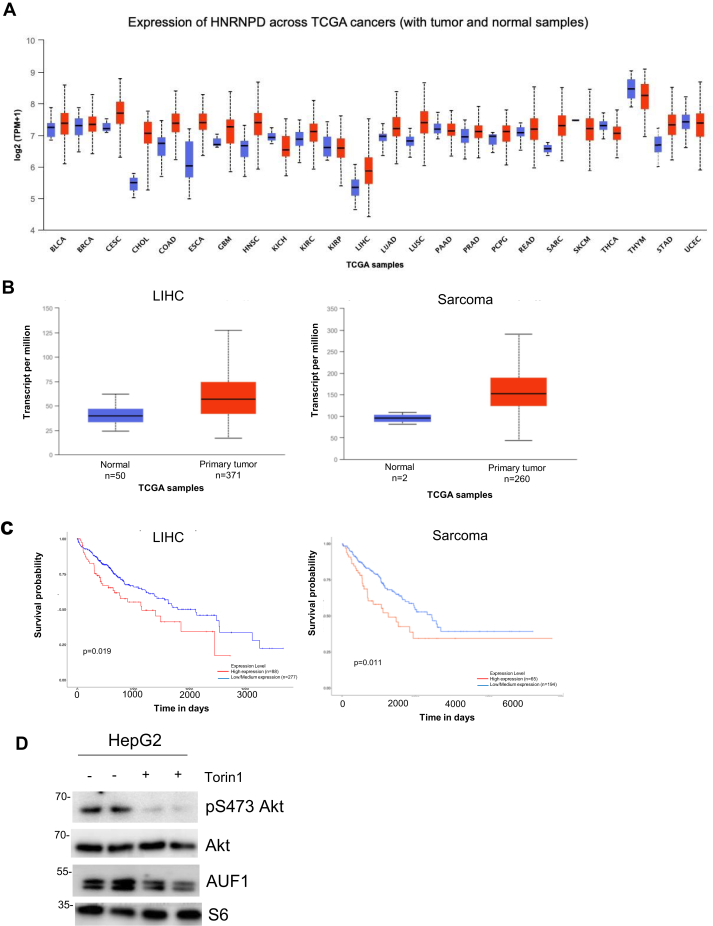
**AUF1 expression is increased in liver tumors.***A* and *B*, UALCAN analysis of expression of AUF1 (hnRNP D) across cancers curated by The Cancer Genome Atlas (TCGA) were compared in tumor *versus* normal samples. *B*, box-whisker *plot* shows in more detail the comparison of normal *versus* tumor samples in liver hepatocellular carcinoma (LIHC) (statistical significance = 1.62E-12) and sarcoma (SARC) (statistical significance = 9.12E-02). *C*, survival plot showing the effects of AUF1 expression level on LIHC and sarcoma (SARC) patients. *p* Value is indicated. *D*, HepG2 cells grown in complete media were treated with vehicle or Torin1 for 1 h. Extracts were subjected to SDS-PAGE and immunoblotting. AUF1, ARE/poly(U)-binding/degradation factor 1; BLCA, bladder urothelial carcinoma; BRCA, breast invasive carcinoma; CESC, cervical squamous cell carcinoma; CHOL, cholangiocarcinoma; COAD, colon adenocarcinoma; ESCA, esophageal carcinoma; GBM, glioblastoma multiforme; hnRNP D, heterogeneous nuclear ribonucleoprotein D; HNSC, head and neck squamous cell carcinoma; KICH, kidney chromophobe; KIRC, kidney renal clear cell carcinoma; KIRP, kidney renal papillary cell carcinoma; LUAD, lung adenocarcinoma; LUSC, lung squamous cell carcinoma; PAAD, pancreatic adenocarcinoma; PCPG, pheochromocytoma and paraganglioma; PRAD, prostate adenocarcinoma; READ, rectum adenocarcinoma; SKCM, skin cutaneous melanoma; STAD, stomach adenocarcinoma; THCA, thyroid carcinoma; THYM, thymoma; UCEC, uterine corpus endometrial carcinoma.

Increased Akt phosphorylation is often a hallmark of cancer (11). We therefore investigated whether AUF1 could be upregulated in tumors with increased Akt phosphorylation. We mined The Cancer Genome Atlas (TCGA) database using UALCAN to compare AUF1 mRNA (hnRNP D) expression in normal *versus* different tumor samples (46). Several types of cancers are associated with increased AUF1 (hnRNP D) gene expression (Fig. 6*A*). However, among these cancers, liver hepatocellular carcinoma and sarcoma displayed a significant increase that is linked to poor patient survival (Fig. 6, *B* and *C*). We then examined the expression of AUF1 in a hepatocellular carcinoma cell line, HepG2. Upon incubation with Torin1, pSer473 Akt phosphorylation was diminished, corresponding with decreased AUF1 expression (Fig. 6*D*). These results are consistent with the effects of Torin1 in downregulating AUF1 expression in HeLa cells (Fig. 5*E*). Together, these findings indicate that AUF1 expression is sensitive to mTOR inhibition in HepG2 cells.

## Discussion

mTORC2 is activated by growth factor/PI3K signaling and nutrient limitation (1, 6). It is required for phosphorylation, optimal activation, and stability of Akt. How mTORC2 signaling is modulated and how mTORC2 mediates Akt phosphorylation remain poorly understood. In the current studies, we found that AUF1, an RBP involved in mRNA degradation and translation, is essential for Akt phosphorylation in the membrane. It is also required for the phosphorylation of GFAT1, a metabolic enzyme that we have previously shown to be modulated by mTORC2 (19, 47). In turn, AUF1 is phosphorylated during conditions that enhance mTORC2 activation. Our findings reveal that in addition to a role for AUF1 in translation and mRNA stability, it modulates mTORC2 signaling.

mTORC2 is necessary for the phosphorylation of Akt at Thr450 and Ser473 (7, 8, 15, 16, 48). Whereas Akt phosphorylation at the activation loop site, Thr308, by PDK1 activates Akt, the phosphorylation at Thr450 and Ser473 allosterically enhances Akt stability and activity, respectively. Here, we have shown that AUF1 is required for phosphorylation of these sites in Akt (Fig. 1). Previous studies in colorectal cancer cells have also demonstrated that downregulation of AUF1 diminishes Akt phosphorylation (49). Importantly, we demonstrate here that AUF1 promotes Akt phosphorylation by facilitating its membrane localization. The loss of AUF1 excludes Akt from the membrane-containing fractions, thus preventing Akt phosphorylation (Fig. 3*A*). Since the phosphorylation of Akt at Ser473 and Thr308 occurs during PI3K activation wherein PDK1 and mTORC2 both localize at the membrane, our findings support that AUF1 facilitates phosphorylation of Akt in the membrane compartment. Hence, in addition to the Akt PH domain, which allows binding to PIP3 that are present in membranes (50), AUF1 is also necessary for this membrane localization to allow Akt phosphorylation. Consistent with the role of AUF1 in facilitating membrane-localized phosphorylation of Akt, AUF1 is also present in the HSP fractions that contain membrane-bound organelles (Fig. 3*A*). Unlike Akt-Ser473 and Akt-Thr308 phosphorylation, the phosphorylation of Akt-Thr450 is not dependent on PI3K but relies on mTORC2 (15, 16). Its phosphorylation occurs during translation as the nascent Akt polypeptide emerges from the ribosome (17). Since both mTORC2 and AUF1 associate with ribosomes (17, 43, 51, 52), AUF1 may promote Thr450 phosphorylation of the nascent Akt chain as part of the translational complex. Further studies are needed to address how AUF1 could promote Akt phosphorylation *via* facilitating localization to specific membrane compartments and/or translational complex.

AUF1 is an RBP with diverse RNA targets and functions (32). Depending on specific targets, it can either positively or negatively regulate mRNA stability. It is also involved in other functions such as translation and miRNA regulation. Our studies reveal that it binds to mRNAs of proteins that are involved in mTORC2 signaling. AUF1 has been reported to modulate Akt signals (49), but our studies demonstrate for the first time that AUF1 binds to Akt mRNA (Fig. 2*A*). The binding to Akt mRNA suggests that it may have a more direct role in modulating Akt translation and co/post-translational phosphorylation by possibly localizing its mRNA to a membrane compartment. However, since AUF1 can also bind to mTOR and rictor mRNAs, as shown previously using PAR-CLIP analysis and to SIN1 mRNA as we demonstrate here (Fig. 2, *B* and *C*), it remains to be further investigated how AUF1 could have a more prevalent role in modulating mTORC2 signaling in general. AUF1 binds to c-Myc mRNA and controls its translation by competing for a common binding site with the translational suppressor TIAR (41). Like mTORC2, c-Myc responds to nutrients and controls glucose and glutamine metabolism (1, 53). Hence, whether AUF1 could control mRNA translation and co/post-translational regulation of nutrient-regulated signalosomes remains to be investigated. Whether AUF1 modulates mTORC2 signaling *via* protein–protein interactions also remains to be addressed. In this regard, AUF1 has been reported to localize at sites of DNA damage independently of its RNA regulatory function (54). Thus, the physical and functional interactions of AUF1 with mTORC2 signaling components warrant further investigation.

We also demonstrate that AUF1 phosphorylation coincides with mTORC2 signaling. First, signals that enhance mTORC2 signaling such as serum restimulation and glucose and/or glutamine withdrawal increase AUF1 phosphorylation. AUF1 has four isoforms that are generated by alternative splicing of the same pre-mRNA. Among these isoforms, p40 and p45 contain exon 2, which encodes an additional 19 amino acids adjacent to RRM1 (Fig. 4*A*), and the protein product of this region includes the phosphosites, Ser83 and Ser87. Ser87 phosphorylation of AUF1 is modulated by PKA and is linked to positive regulation of AUF1 activity (43, 55, 56). Since AUF1 phosphorylation is enhanced by signals that increase mTORC2 signaling, this would suggest that PKA could couple signals from mTORC2 to AUF1. How PKA could mediate mTORC2 signals is poorly understood and thus remains to be further studied particularly in mammalian cells (57, 58, 59, 60). Phosphorylation of Ser83 is mediated by glycogen synthase kinase 3 (GSK3) and occurs only when Ser87 is phosphorylated (55). Ser83 phosphorylation is believed to repress the transactivation function of AUF1. Speculatively, this repression may occur as mTORC2 signals diminish. In support of this notion, we found that AUF1 phosphorylation is sensitive to nutrient levels and mTOR activity. When phosphorylated, AUF1 facilitates Akt phosphorylation and activation. GSK3 is a substrate of Akt. The Akt-mediated GSK3α/β phosphorylation at Ser21/9 inhibits GSK3 to consequently promote cell survival and proliferation (11). Hence, it is possible that increased Akt activation could further boost AUF1 phosphorylation at Ser87 and activity by repression of GSK3.

Increased mTORC2/Akt signaling is often found in tumors. The increased phosphorylation of Akt is used as a hallmark of increased proliferation in cancer cells (11). mTOR and Akt inhibitors are currently undergoing clinical trials to treat various cancers and other metabolism-related disorders (4, 61). AUF1 is overexpressed in different tumors (49, 62, 63, 64, 65) (Fig. 6, *A* and *B*). Its increased expression in human liver carcinoma, sarcoma, and colorectal cancer is associated with decreased survival or poor prognosis (Fig. 6*C*) (49). We have shown here that inhibition of mTOR in the hepatocarcinoma cell line, HepG2, diminishes AUF1 expression and Akt phosphorylation (Fig. 6*D*). Our findings reveal that AUF1 could serve as a potential target for modulating mTORC2/Akt signaling. Further studies to understand how AUF1 can specifically control Akt phosphorylation would be important for development of more effective therapeutics against cancer and metabolic disorders.

## Experimental procedures

### Materials

AUF1 rabbit polyclonal antibody was previously described (66); pAUF1 antibody (rabbit polyclonal against exon 2-encoded, 16-amino acid peptide spanning pSer83 and pSer87). pSer243 GFAT1 (47); GFAT1 (Abcam; catalog no.: ab125069); SIN1 (Abcam; catalog no.: ab71152); actin (Santa Cruz Biotechology; catalog no.: sc-53029); and calnexin (Santa Cruz Biotechnology; catalog no.: sc-6465). All other antibodies were obtained from Cell Signaling Technology with the following catalog numbers: pThr308Akt (13038); pThr450Akt (9267); pSer473-Akt (4060); Akt (9272); pThr389 S6K1 (9234); S6K1 (9202); pSer240/244 S6 (2215); S6 (2317); rictor (9476); mTOR (2983); PDK1 (3062); PTEN (9559); and HKII (2106). Torin1 was purchased from Tocris.

### Plasmids, transfection, and IP

The following AUF1-related plasmids were described previously: plasmids expressing scrambled (control) shRNA (shCTRL) or shRNA directed against all four AUF1 isoforms (shAUF1) and plasmids expressing individual AUF1 isoforms refractory to shAUF1 (hereafter designated as AUF1^R^) (45). Plasmids expressing shAUF1-refractory (R), phosphomimetic mutations (dual Ser83 and Ser87 to Asp [D]) and shAUF1-refractory (R) phospho-resistant mutations (dual Ser83 and Ser 87 to alanine [A]) (67). siRNA for AUF1 (hs.RI.HNRNPD.13.1) and scrambled control were obtained from IDT. Plasmids expressing WT myristoylated Akt (WT Myr-Akt) or KD myristoylated Akt (KD Myr-Akt) were previously described (15).

THP-1 cells were stably transfected using Effectene Transfection Reagent (Qiagen) with plasmids expressing either shCTRL or shAUF1, and neomycin-resistant cells were pooled for further experiments, as described previously (45).

Transient transfections of plasmid DNAs into HeLa cells were performed as previously described (67). For cotransfection of shAUF1 or shCTRL and Myr-Akt constructs, HeLa cells were grown in complete media until about 60% confluence in a 6-well plate. Prior to transfections, cells were resuspended in fresh complete or starvation media as indicated. About 0.5 to 2 μg of plasmid DNA that was preincubated with Lipofectamine 2000 (Thermo Fisher Scientific) was added per manufacturer’s directions. About 24 h post-transfection, cells were resuspended in corresponding media followed by cell lysis.

mRNP IPs and qRT–PCR (mRNP immunoprecipitation–qRT–PCR) assays were performed with HeLa and THP-1 cell lysates and either nonimmune rabbit serum (Sigma) or AUF1 antibody as previously described (68). RNAs were purified from precipitations and analyzed by qRT–PCR using the following primer sets:

Akt (Forward: 5′aaaaaggtctccgctggcgctgagattgtgtcagc; Reverse: 5′aaaaaggtctcccagcgaagcgggcccggtcctc), SIN1 (Forward: 5′gtattagaagacgctcaaacgcagctcaaagattagaacga; Reverse: 5′tcgttctaatctttgagctgcgtttgagcgtcttctaatac), GFAT1 (Forward: 5′ccccagtcccacagaagtat; Reverse: 5′aactgacagcattggctttg), rictor (Forward: 5′ctaggtggcattgacattcagc; Reverse: 5′ctaggaaacaaggaagcattcag), mTOR (Forward: 5′ccaagcttatgcttggaaccggacctgcc; Reverse: 5′aaccgcggccagaaagggcacca); c-Myc (Forward: 5′acgaaactttgcccatagca; Reverse: 5′gcaaggagagcctttcagag), and GAPDH (Forward: 5′gattgttgccatcaacgacc; Reverse: 5′ccatggtggtgaagacacca).

Protein co-IP was performed on cells lysed with CHAPS lysis buffer (40 mM Hepes, 2 mM EDTA, 0.38% CHAPS, and 150 mM NaCl) as described previously (47). Precleared extracts were incubated with SIN1 antibody, and co-IPs were recovered using Protein G agarose beads.

### Cell culture, lysis, and immunoblotting

MEFs, HeLa, or THP-1 cells were seeded at around 150,000 to 250,000 cells/ml in complete media: Dulbecco's modified Eagle's medium (Sigma; catalog no.: D6546) containing 10% fetal bovine serum (FBS), 2 mM glutamine (Gibco; catalog no.: 25030-164), and penicillin/streptomycin (Gibco; catalog no.: 15140-122). HepG2 was cultured in Eagle’s minimum essential medium with l-Gln (American Type Culture Collection; catalog no.: 30-2003) containing 10% FBS. After culturing for 20 to 24 h (to reach 70–80% confluency), the cells were washed in PBS and resuspended in either fresh complete media or starvation media (glucose starvation media, Corning, catalog no.: 17-207-CV; glutamine starvation media, Corning, catalog no.: 15-017-CV) as described previously (19). The following were also added to resuspension media as denoted: 10% dialyzed FBS (Hyclone; catalog no.: SH30079.03); 25 mM glucose, or 2 mM glutamine. Cells were harvested with radioimmunoprecipitation assay lysis buffer (50 mM Tris–HCl; pH 8.0, 100 mM NaCl, 5 mM EDTA, 0.2% SDS, 0.5% sodium deoxycholate, and 1.0% Triton X-100), containing protease and phosphatase inhibitors. Protein concentrations were determined by Bradford analysis, and 20 to 30 μg of sample proteins was subjected to SDS-PAGE. Proteins were transferred onto Immobilon polyvinylidene fluoride membrane (Millipore). Membranes were incubated with primary antibodies overnight in PBS/Tween with 0.5% nonfat dry milk. Membranes were washed in PBS/Tween. After incubation with the secondary antibody, blots were washed again in PBS/Tween. Images were visualized by SuperSignal ECL detection kit (Thermo Fisher Scientific) and captured using Amersham Biosciences Imager 600 (GE Healthcare).

### Cellular fractionation

Cells were lysed in CHAPS lysis buffer and centrifuged at 14,000 rpm. Supernatant is designated as the cytosolic fraction. The pellets, which consist of membrane-containing compartments, were further passed through a 27G1/2 needle 15 times, and samples were centrifuged at 1000*g* for 10 min at 4 °C. The low-speed pellet was resuspended in radioimmunoprecipitation assay buffer, and supernatant was ultracentrifuged at 65,000 rpm for 1 h at 4 °C. The resulting HSP was resuspended in HSP buffer (10 mM Tris–HCl, pH7.5, 75 mM NaCl, 0.5 mM EDTA, 0.5 mM EGTA, and 0.5% Triton X). All buffers were supplemented with protease and phosphatase inhibitors.

### AUF1 expression analysis from TCGA database

AUF1 mRNA expression from normal *versus* different tumor samples from the TCGA database was analyzed using http://ualcan.path.uab.edu as previously described (46). Statistical significance provided were estimated by Student’s *t* test. Kaplan–Meier plot shows the effect of gene expression on patient survival. The significance of survival impact as provided was measured by log rank test.

## Data availability

All data are available in the main text or the supporting information. Research materials used in the studies are available upon request from the authors.

## Supporting information

This article contains supporting information.

## Conflict of interest

The authors declare that they have no conflicts of interest with the contents of this article.
