# Supplementary material for: The RNA-binding protein AUF1 facilitates Akt phosphorylation at the membrane
Source: J Biol Chem. 2022 Aug 27;298(10):102437. doi: 10.1016/j.jbc.2022.102437 (PMC9513781; doi:10.1016/j.jbc.2022.102437)
Supplement: Supplementary Figures [file mmc1.pdf]

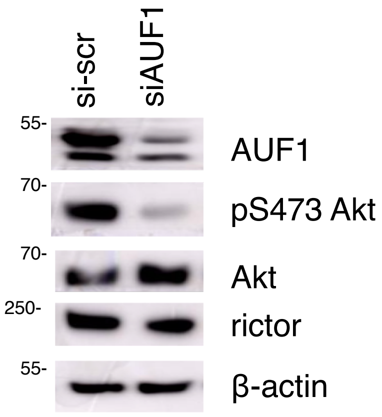

Fig. S1. Phosphorylation of Akt is abolished upon AUF1 knockdown. HeLa cells were transiently transfected with scrambled siRNA (si-scr) or siAUF1. Cell extracts were subjected to SDS-PAGE and immunoblotting.

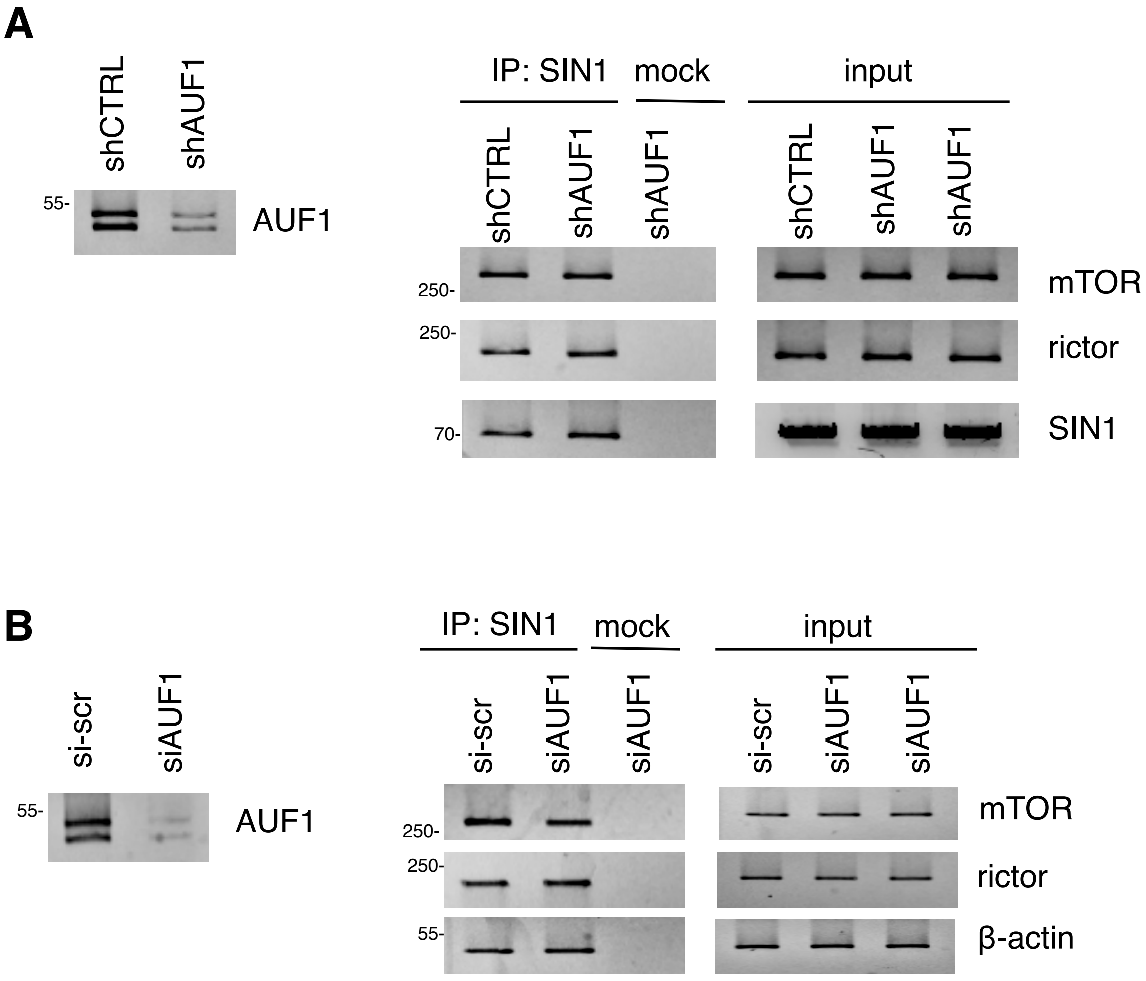

Fig. S2. mTORC2 complex integrity is not compromised during knockdown of AUF1 expression.

HeLa cells were transiently transfected with plasmids expressing shCTRL or shAUF1 (A) or either scrambled siRNA (si-scr) or siAUF1 (B). Cell extracts were subjected to immunoprecipitation using SIN1 antibody. Co-immunoprecipitates and total extracts (input) were fractionated by SDS-PAGE followed by immunoblotting.
